# Fatty Acid Composition and Cytotoxic Activity of Lipid Extracts from *Nannochloropsis gaditana* Produced by Green Technologies

**DOI:** 10.3390/molecules27123710

**Published:** 2022-06-09

**Authors:** Natalia Castejón, Doris Marko

**Affiliations:** Department of Food Chemistry and Toxicology, Faculty of Chemistry, University of Vienna, Währinger Str. 38, 1090 Vienna, Austria; doris.marko@univie.ac.at

**Keywords:** microalgae, omega-3 fatty acids, lipid recovery, eco-friendly approaches, cytotoxicity, bioactive compounds, biological activity

## Abstract

Microalgae are alternatives and sustainable sources of omega-3 long chain-polyunsaturated fatty acids (LC-PUFA). However, the eco-friendly extraction of these bioactives remains unexplored. In this work, the use of enzyme-based methods in combination with ultrasounds was evaluated as green approaches to extract the omega-3 lipids from *Nannochloropsis gaditana*. Three commercial enzymatic solutions (Viscozyme^®^ L, Celluclast^®^ 1.5 L, and Saczyme^®^) were investigated, and results were compared with the traditional Folch method. A promising extraction approach was developed by using Saczyme^®^, achieving a lipid yield of 25.7% ± 0.5, comparable to the traditional method (27.3% ± 0.7) (*p* > 0.05). Similar omega-3 content was found by GC–MS analysis for both lipid extracts (30.2% ± 2.4 and 29.3% ± 0.8 for the green and the traditional method, respectively), showing that the green approaches did not affect the fatty acid profile. Moreover, the cytotoxic activity of produced lipids was assessed by comparing human colon cancer cells (HCT-116) and epithelial nontumorigenic immortalized cells (HCEC-1CT). Results suggest that the lipid extracts have a selective effect, reducing the viability of the colon carcinoma cells but not the nontumorigenic cells. Thus, this study provides new eco-innovative approaches for extracting the omega-3 LC-PUFA from microalgae with promising biological properties.

## 1. Introduction

In recent years, the demand for novel bioactive compounds with potential health benefits has experienced an outstanding increase. Today’s consumers have become more environmentally conscious, preferring natural products and considering the impact of food choices on our planet. These concerns have turned the scientific community to explore the use of naturally occurring species, including microalgae, for sustainable and eco-friendly sources of food ingredients and products [1,2].

Microalgae are unicellular photosynthetic microorganisms that convert sunlight, water, and carbon dioxide to algal biomass. Along with the prokaryotic cyanobacteria, often called blue-green microalgae, they provide a wide range of high-valuable compounds, such as carotenoids, phenolic compounds, peptides, sulfated polysaccharides, and valuable lipids. [3,4]. Moreover, microalgae are the primary producers of the omega-3 long-chain polyunsaturated fatty acids (LC-PUFA), namely eicosapentaenoic (EPA) and docosahexaenoic (DHA) acids. EPA and DHA are well accepted as essential components for human health, having beneficial effects on brain development and mitigating different pathological conditions [5]. Compared to terrestrial crop plants, microalgae present advantages as omega-3 LC-PUFA sources, such as higher growth rates, superior growth performance and productivity, and lesser land requirements [6]. Thus, microalgae are crucial sources for meeting population needs in terms of omega-3 PUFA and more complex lipids, including glycolipids and phospholipids.

The extraction of lipids containing omega-3 LC-PUFA from microalgae remains a challenge. One of the most critical points in this process is the selection of the solvent and the extraction technique. Microalgae have a dense and firm cell wall, and the extraction efficiency of the omega-3 lipids is limited by the rigidity of the microalgae matrix [7]. Consequently, a suitable pre-treatment and extraction approach should be carefully selected to recover the valuable lipids from microalgae and maintain their biological activity [8].

*Nannochloropsis* spp. are one of the most promising species for producing high-value lipids containing omega-3 PUFA, specifically EPA [9,10]. Different advanced extraction techniques have been reported in the literature such as subcritical and supercritical fluids, ultrasound, microwaves, pulsed electric energy, and enzymatic methods, aimed at the degradation of the complex cell walls of *Nannochloropsis* spp. [11]. Among these approaches, enzyme-based methods showed that they were effective, nontoxic, and low in energy consumption procedures when improving the extraction of intracellular compounds [12,13,14]. Moreover, the combination of different methods could open new alternatives with reduced time, consumed energy, and less use of toxic solvents. In this regard, enzyme-based alternatives in combination with a widely applied technique in food processing like ultrasounds could be the key to emerging eco-innovative extraction approaches.

On the other hand, the evaluation of the cytotoxic activity of natural extracts has an important role in the development of novel ingredients with potential application in the food and nutraceutical industry. Only a few studies have reported the cytotoxic and/or biological properties of *Nannochloropsis gaditana* extracts by in vitro cell-based assays. For instance, Letsiou et al. [15] investigated the skin protective effects of *Nannochloropsis gaditana* extract on human dermal fibroblasts. In a recent study, Carrasco–Reinado et al. [16] reported the antiproliferative activity of a recombinant protein against tumor cell lines. Moreover, Ávila–Román et al. evaluated the effect of oxylipins isolated from *Nannochloropsis gaditana* against human cancer cell lines and their impact on ATP levels [17]. The same group also investigated the activity of these oxygenated compounds as TNF-α and NFκB inhibitors [18,19]. However, to the best of our knowledge, this is the first time that the cytotoxic activity of omega-3 lipid extracts from *Nannochloropsis gaditana* is assessed against human colon cancer and nontumorigenic cells. Additionally, the impact of eco-friendly approaches on the biological activities of produced extracts remains unexplored.

Therefore, the present study aimed to investigate the use of enzyme-based alternatives in combination with ultrasounds as novel methods to produce high-quality lipid extracts from *Nannochloropsis gaditana*. The effect of eco-friendly approaches on the fatty acid profile was also evaluated by gas chromatography–mass spectrometry (GC–MS). Moreover, the cytotoxicity activity of produced microalgal lipids was investigated by a spectrum of in vitro human cell-based assays as a first attempt to evaluate the potential of these valuable lipids in health and food applications.

## 2. Results and Discussion

### 2.1. Effect of Ultrasound-Assisted Extraction on the Lipid Recovery from Nannochloropsis gaditana

**Figure 1:**
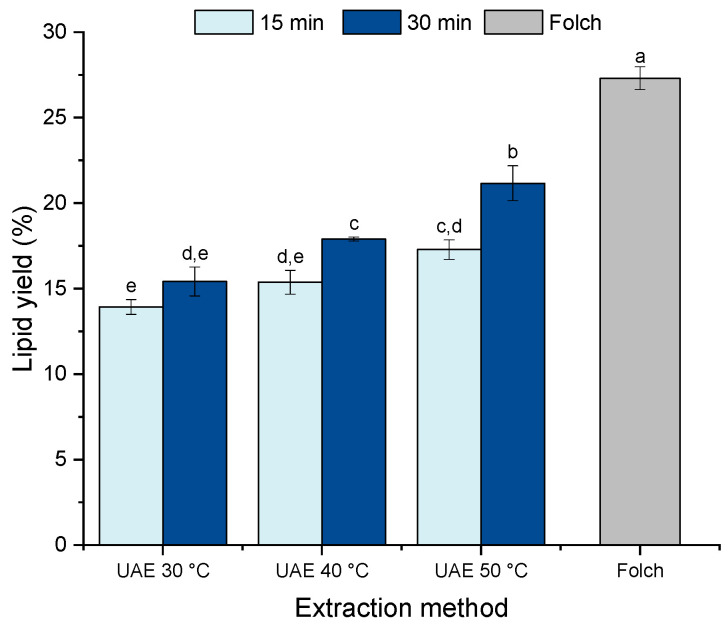
Effect of ultrasound-assisted extraction (UAE) process parameters (time and temperature) on lipid recovery. Results are expressed as a percentage of dry weight. Error bars denote the standard deviation of three independent extractions (n = 3). Different letters indicate statistically significant differences at *p* < 0.05 (one-way ANOVA with post-hoc Tukey, a–e).

In a first step, ultrasound-assisted extraction (UAE) and ethanol were evaluated as green alternatives to the traditional Folch method. Figure 1 shows the effects of UAE treatment time (15 and 30 min) and temperature (30, 40, and 50 °C) on lipid yield in comparison with the traditional extraction method. Significant differences were found in the lipid yield between the different UAE conditions investigated, ranging from 14 to 21%. Increasing the extraction time from 15 to 30 min improved the lipid recovery for all temperatures investigated except for the extraction carried out at 30 °C, wherein the extraction time did not affect the lipid yield (*p* > 0.05). For the extraction time of 30 min, raising the temperature from 30 °C to 50 °C exhibited an increasing trend in lipid yield (*p* < 0.05), showing a notable effect of the temperature on this process. Despite the positive effect of the extraction temperature, the conditions tested were not suitable for archiving the lipid recovery obtained by the traditional Folch method (27.3% ± 0.7).

The use of ultrasounds has been recognized as an efficient extraction technique to disrupt the cell wall of different microalgae (for example *Chlorella vulgaris* [20], *Schizochytrium limacinum* [21], *Chlamydomonas reinhardtii* [21], *Isochrysis galbana* [22,23], *Desmodesmus* sp. [24]. etc.); however, for the specific case of *Nannochloropsis gaditana* and under the conditions tested, it was not enough to achieve a complete lipid recovery. The presence of a thick and rigid cell wall in *Nannochloropsis gaditana* composed of a bilayer structure with a cellulose-based inner layer protected by an outer hydrophobic algaenan layer could explain the inefficiency of the ultrasounds and ethanol [25]. For that reason, aiming to find efficient alternatives to disrupt the microalgal cell wall, enzyme-based alternatives in combination with ultrasounds were further evaluated.

### 2.2. Enzyme-Based Approaches as Green Alternatives to the Conventional Extraction of Lipids from Nannochloropsis gaditana

**Figure 2:**
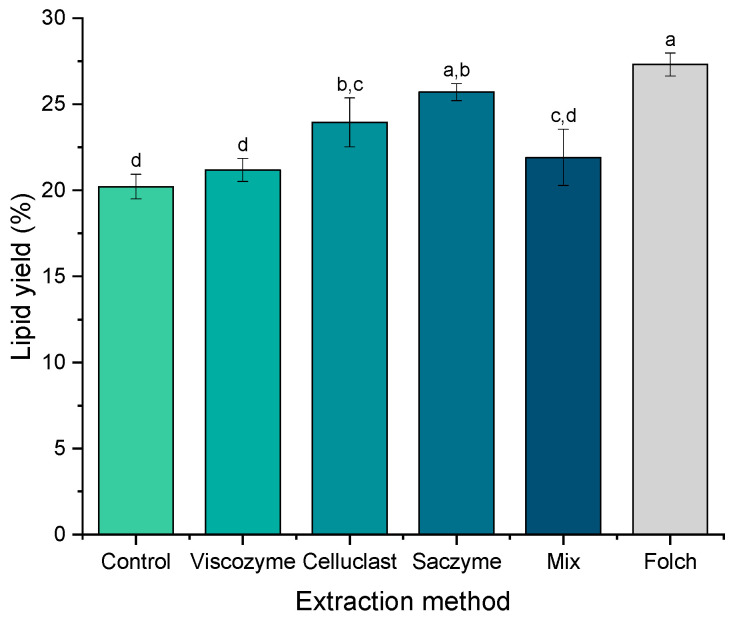
Lipid extraction yield using enzyme-based alternatives with different commercial enzymatic solutions: Viscozyme^®^ L, Celluclast^®^ 1.5 L, Saczyme^®^ Yield, and a mix of the enzymatic solutions (1:1:1 *v*/*v*). A control assay was done without enzymatic solution (control). Results are expressed as a percentage of dry weight. Error bars denote the standard deviation of three independent extractions (n = 3). Different letter indicates statistically significant differences at *p* < 0.05 (one-way ANOVA with post-hoc Tukey, a–d).

In a second step, the use of different enzymatic solutions was investigated to develop efficient and sustainable alternatives to extract the omega-3 lipids from *Nannochloropsis gaditana.* The alternative approaches are based on the application of three commercial enzymatic solutions (Viscozyme^®^ L, Celluclast^®^ 1.5 L, Saczyme^®^ Yield) and a mix of them (1:1:1 *v*/*v*) (Figure 2).

Interesting results were found during the use of the enzyme-based alternatives. The lipid recovery ranged from 20 to 26% depending on the enzymatic solution applied. Unexpectedly, the use of Viscozyme and the enzymatic mix did not achieve an improvement of the lipid yield (21.1% ± 0.6 and 21.9% ± 1.6, for Viscozyme and the mix, respectively) in comparison with the control experiment (20.2% ± 0.7) (no enzymatic solution added) (*p* > 0.05). These results were in contrast with those of Blanco–Llamero et al., who reported a synergic effect when different hydrolytic enzymes were used simultaneously for the same microalgae species [26].

A different behavior was exhibited for the enzymatic solution Saczyme, which seems to be the most effective strategy to extract the omega-3 lipids from *Nannochloropsis gaditana*. The lipid yield using Saczyme was 25.7% ± 0.5, comparable to the result obtained with the traditional Folch method (27.3% ± 0.7) (*p* > 0.05). Saczyme is an enzymatic blend containing glucoamylase, acid amylase, and cellulase. It has been recently used for the valorization of lignocellulosic residues from the olive oil industry [27] or the valorization of industrial bark residues [28], among other applications in the food and beverage industry according to the manufacturer. However, to our knowledge, this is the first time that Saczyme is efficiently used to extract the lipids from microalgae. Thus, a new and promising green extraction approach was developed by using UAE with the commercial preparation Saczyme, achieving a lipid yield comparable to the traditional method.

Other green extraction methods have been used for the lipid extraction of *Nannochloropsis gaditana*. For instance, Molino et al. [29] and Taher et al. [30] reported the use of supercritical CO_2_ as an emerging technology for the extraction of these compounds. Ho et al. [31] used a similar strategy to extract the omega-3 EPA but using subcritical water. The use of pressurized liquids has also been reported by several authors [10,32,33]. Moreover, in a recent study, Jiménez Callejón et al., [34] investigated the simultaneous extraction and fractionation of polar lipids by using supercritical and pressurized liquids. The use of these advanced technologies minimizes the energy costs and the environmental impact. However, the price and initial investment of the main equipment (supercritical fluid or pressurized liquids extractors) need to be considered, and ultrasounds or enzyme-based approaches could result in a lower economic cost. Heredia et al. [35] and Jiménez Callejón et al. [36] also aimed to develop energy-efficient processes for recovering the lipids from *Nannochloropsis gaditana*, nevertheless, by using the wet paste biomass. Microalgal wet pastes usually contain more than 70–80% water, which makes the extraction process more challenging compared to the use of dry biomass (as in the present study).

### 2.3. Fatty Acid Profile of Lipid Extracts from Nannochloropsis gaditana Produced by Green Extraction Alternatives

One of the objectives of the present study was to evaluate the influence of the green extraction approaches on the fatty acid profile of *Nannochloropsis gaditana*. For that reason, all microalgal lipid extracts produced in this study were analyzed by GC–MS. Table 1 shows the fatty acid composition (as percentage of total fatty acids) of lipid extracts obtained from *Nannochloropsis gaditana* comparing the different enzymatic solutions with the traditional Folch method. The fatty acids identified were lauric acid (12:0), myristic acid (14:0), palmitic acid (16:0), palmitoleic acid (16:1 cis-9), stearic acid (18:0), vaccenic acid (18:1 cis-7), oleic acid (18:1 cis-9), linoleic acid (18:2 cis-cis-9,12), arachidonic acid (20:4 cis-5,8,11,14), and eicosapentaenoic acid (20:5 cis-5,8,11,14,17).

The major fatty acids were palmitic, palmitoleic, and the omega-3 EPA, which were detected in ranges of 18.3% ± 1.3 to 23.0% ± 0.8, 22.9% ± 0.8 to 26.1% ± 0.6, and 29.3% ± 0.8 to 33.8% ± 1.1, respectively. Additionally, *Nannochloropsis gaditana* lipid extracts were characterized by a high percentage of PUFA (39.7–47.0%) and a low n-6/n-3 ratio (0.3), showing the excellent nutritional properties of this microalgae. These results are in line with previous studies reporting a comparable fatty acid profile [10,32,37,38].

The fatty acid compositions of extracted lipids were very similar in percentage regardless of the enzymatic solution used. Only slight variations were found in certain fatty acids, for instance, palmitic and palmitoleic acid. Regarding the omega-3 content, no significant differences were found between the different techniques used (*p* > 0.05). The overall impact of the enzyme-based approaches on the lipid composition was negligible Previous studies have also reported that there were no significant changes in the fatty acid composition using different extraction methods and experimental conditions [39]. Thus, we can conclude that the green extractions techniques developed in this work are efficient alternatives to extracting the high-value lipids of *Nannochloropsis gaditana* without affecting the fatty acid profile.

### 2.4. Cytotoxic Activity of Lipid Extracts from Nannochloropsis gaditana

To evaluate the cytotoxic properties of *Nannochloropsis gaditana* lipid extracts, a spectrum of in vitro human cell-based assays was performed. Cell viability was assessed by metabolic activity (CellTiter-Blue (CTB) assay), protein content (sulforhodamine B (SRB) assay), and lactate dehydrogenase (LDH) release (CyQuant LDH assay). The lipid extract obtained with the enzymatic solution Saczyme was selected because it was the most effective strategy among the enzyme-based approaches investigated and was compared with the lipid extract produced by the traditional method. The cytotoxic activity of both lipid extracts was assessed by comparing human colon cancer cells (HCT-116) and epithelial nontumorigenic immortalized cells (HCEC-1CT). The lipid extracts were incubated for 24 h in a range of concentrations from 0.1 µg/mL to 200 µg/mL.

**Figure 3:**
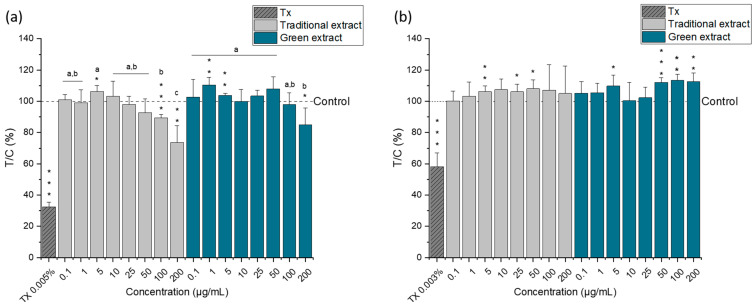
Cell viability of HCT-116 cells (**a**) and HCEC-1CT (**b**) after 24 h incubation with different concentrations of the omega-3 lipid extracts produced by the traditional method (traditional extract) and the green method by using Saczyme (green extract) measured by CTB assay. Triton X (Tx; 0.005 or 0.003%) was used as positive control. Results are presented as mean + SD normalized to DMSO solvent control (0.5%; T/C in %) (n = 6). Statistical differences compared to the DMSO solvent control were calculated with a one-sample Student’s *t*-test (* *p*, ** *p*, *** *p* < 0.05, 0.01, 0.001). Significant differences among the test concentrations were calculated with one-way ANOVA (*p* < 0.05, a–c).

According to the CTB assay (Figure 3), both lipid extracts significantly reduced the metabolic activity of the tumorigenic cell line (HCT-116) at the highest concentrations tested (100 and 200 µg/mL) (Figure 3a). Surprisingly, *Nannochloropsis gaditana* lipid extracts did not alter the metabolic activity of HCEC-1CT cells—i.e., the nontumorigenic cells (Figure 3b). These results suggest that the omega-3 lipid extracts from *Nannochloropsis gaditana* have a selective cytotoxic effect, acting preferably against the tumor cell line, but appear not to affect the nontumor cells.

**Figure 4:**
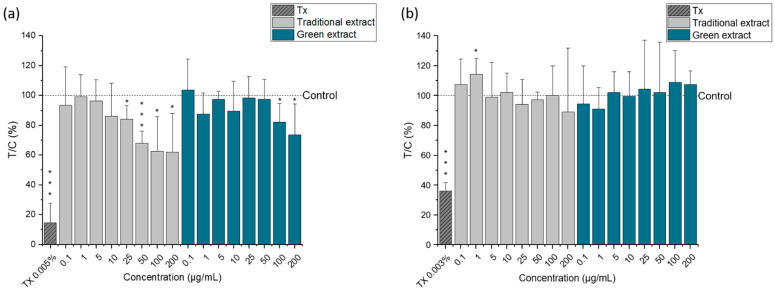
Cell viability of HCT-116 cells (**a**) and HCEC-1CT (**b**) after 24 h incubation with different concentrations of the omega-3 lipid extracts produced by the traditional method (traditional extract) and the green method by using Saczyme (green extract) measured by SRB assay. Triton X (Tx; 0.005 or 0.003%) was used as positive control. Results are presented as mean + SD normalized to DMSO solvent control (0.5%; T/C in %) (n = 5). Statistical differences compared to the DMSO solvent control were calculated with a one-sample Student’s *t*-test (* *p*, *** *p* < 0.05, 0.01, 0.001). Significant differences among the test concentrations were calculated with one-way ANOVA (*p* < 0.05, a–c).

A similar trend of cell proliferation was observed in the SRB assay (Figure 4). The protein content was significantly reduced in HCT-116 cells treated with concentrations above 25 µg/mL for the lipid extract obtained with the traditional method (Figure 4a) and above 100 µg/mL for the lipid extract produced by the green strategy (Figure 4b). In this case, the cytotoxic effect appears to be more evident for the lipid extract produced by the traditional method. Nonetheless, the protein content of HCEC-1CT cells was not affected regardless of the extract type or concentration used.

**Figure 5:**
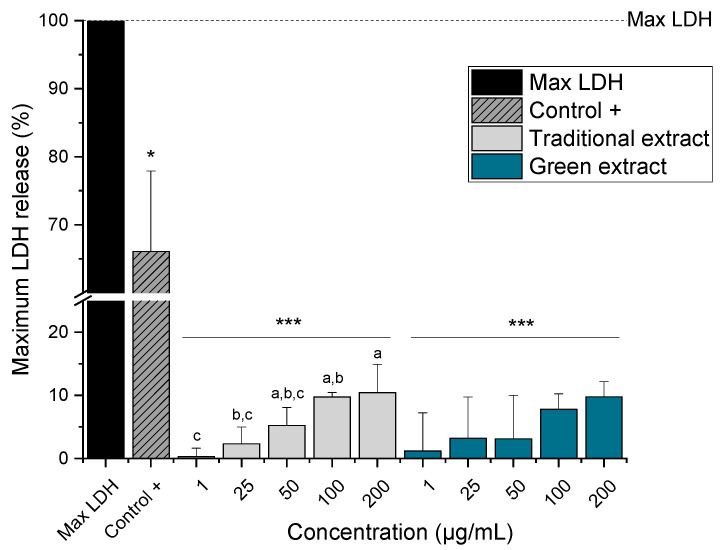
Release of lactate dehydrogenase (LDH) from HCT-116 cells after 24 h incubation with different concentrations of the omega-3 lipid extracts produced by the traditional method (traditional extract) and the green method by using Saczyme (green extract). LDH positive control (control +) used was provided by the kit. Maximum LDH release was determined by lysing the cells by using Triton-X100 for 45 min. Results are presented as mean + SD normalized to the maximum LDH (n = 3). Statistical differences compared to the maximum LDH were calculated with a one-sample Student’s t-test (* *p*, *** *p* < 0.05, 0.01, 0.001). Significant differences among the test concentrations were calculated with one-way ANOVA (*p* < 0.05, a–c).

Moreover, for selected experimental conditions (HCT-116 cell line and selected concentrations: 1, 25, 50, 100, and 200 µg/mL), the LDH release assay was also performed to investigate potential effects on cell membrane integrity (Figure 5). 

LDH is a soluble cytoplasmic enzyme present in nearly all cells, and it is released into the extracellular space when the cell membrane is damaged [40]. Significant LDH release was observed for both lipid extracts, being 10.4% ± 4.5 and 9.8% ± 2.4, the highest LDH level detected for the lipid extract produced by the traditional and green methods, respectively. Moreover, a significant concentration-dependent increase in LDH release was observed for cells treated with the lipid extract produced with the traditional method (*p* < 0.05). Unfortunately, this trend was not observed for the green extract because no significant differences were found between the different concentrations tested.

The findings of the present work are supported by previous studies reporting that omega-3 PUFA causes selective cytotoxicity towards cancer cells with little or no toxicity on normal cells [41,42]. EPA and DHA have been described to induce apoptosis in vitro in tumor cell lines derived from a wide range of tumors, including colorectal carcinoma [43,44,45], esophageal [46], pancreatic cancer [47], and lung cancer [48], among others. The differences between the two lipid extracts in terms of cytotoxic effects exhibited by the SRB and LDH assay could be related to the composition of the extract. Because the Folch and the enzyme-based method are based on different extraction mechanisms, the lipid composition could differ between them. Further analyses regarding the lipidomic composition of the extracts are currently being investigated in our laboratory to clarify the different behaviors observed.

**Figure 6:**
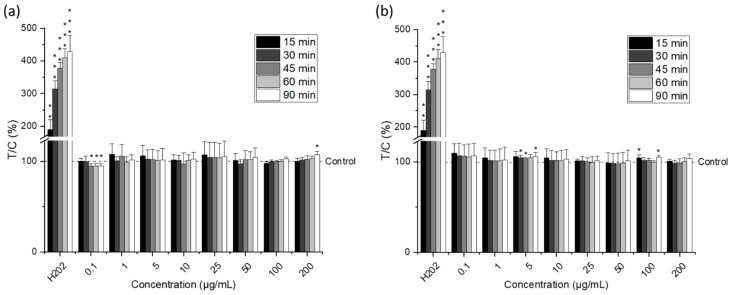
ROS levels in HCT-116 cells after different incubation times (15, 30, 45, 60, and 90 min) with different concentrations of the omega-3 lipid extracts produced by the traditional method (**a**) and the green method by using Saczyme (**b**) measured by the dichlorofluorescein (DCF) assay. Hydrogen peroxide (H_2_O_2_) was used as positive control. Results are presented as mean + SD normalized to DMSO solvent control (0.5%; T/C in %) (n = 6). Statistical differences compared to the DMSO solvent control were calculated with a one-sample Student’s *t*-test (* *p*, ** *p*, *** *p* < 0.05, 0.01, 0.001).

Additionally, in a first attempt to understand the cytotoxicity mechanism of the microalgal extracts in the tumorigenic cells, the effect of omega-3 lipid extracts on cellular ROS levels of HCT-116 cells was investigated with the DCF assay (Figure 6).

**Figure 7:**
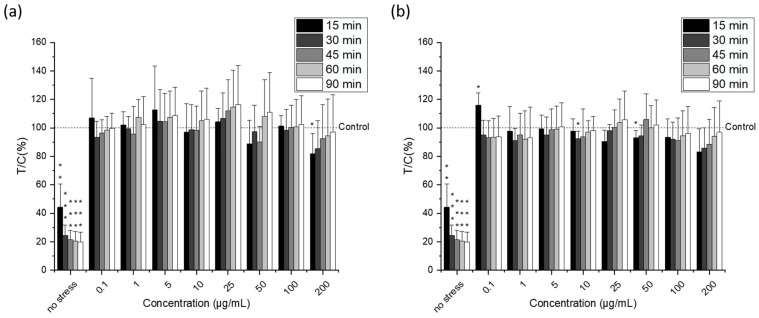
H_2_O_2_-induced ROS-levels in HCT-116 cells after incubation for 24 h with different concentrations of the omega-3 lipid extracts produced by the traditional method (**a**) and the green method by using Saczyme (**b**) measured by the protective dichlorofluorescein (pDCF) assay. Cells were stressed for 90 min with 1 mM H_2_O_2_ whereas fluorescence intensity was measured at different times (15, 30, 45, 60, and 90 min). Results are presented as mean + SD normalized to DMSO solvent control (0.5%; T/C in %) (n = 6). Statistical differences compared to the DMSO solvent control were calculated with a one-sample Student’s *t*-test (* *p*, ** *p*, *** *p* < 0.05, 0.01, 0.001).

Different studies have shown that the accumulation of certain PUFAs leads to increased lipid peroxidation and the generation of intracellular radical species that may be toxic to cancer cells [42,47,49,50]. However, in this study and under the conditions tested, the incubation of HCT-116 cells with the microalgal lipid extract did not result in a significant increase in intracellular ROS levels regardless of the concentration or type of extract used (*p* > 0.05). Moreover, a modified version of the DCF assay, with an extended incubation period (24 h) to allow gene expression, was used to evaluate the ability of microalgal lipids to strengthen the antioxidant defense of cells and thus offer protection from H_2_O_2_-induced ROS production (Figure 7); nevertheless, no significant effects were detected. Then, further in-depth studies are needed to elucidate the mechanisms responsible for the cytotoxicity of *Nannochloropsis gaditana* lipid extracts in tumor cells.

## 3. Materials and Methods

### 3.1. Materials

The lyophilized microalgal biomass of *Nannochloropsis gaditana* (batch L3250520) was purchased from Cianoalgae SI (Gipuzkoa, Spain). Enzymatic solutions (Viscozyme^®^ L, Celluclast^®^ 1.5 L, Saczyme^®^ Yield) were kindly donated by Novozymes A/S (Bagsvaerd, Denmark). The main components of the enzymatic preparations and their activities are reported in Table 2.

Chloroform, methanol, and ethanol were purchased from Fisher Scientific GmbH (Wien, Austria). Sodium hydrogen carbonate, potassium hydroxide, Triton X, and dimethylsulfoxide (DMSO) were purchased from Carl Roth (Karlsruhe, Germany). Cell culture media and respective supplements were obtained from Gibco Thermo Fisher Scientific (Waltham, MA, USA) and Szabo Scandic (Vienna, Austria). CellTiter-Blue (CTB) 10× concentrate was purchased from Promega (Waldorf, Germany). Sulforhodamine B sodium salt (SRB) and 2′,7′-dichlorofluorescin diacetate (DCFH-DA) were purchased from Sigma-Aldrich (München, Germany). Invitrogen CyQuant LDH Cytotoxicity Assay Kit was bought from Thermo Fisher Scientific (Waltham, MA, USA). Fatty acid methyl esters standard (Supelco 37 FAME Mix) was from Supelco (Bellefonte, PA, USA).

### 3.2. Lipid Extraction of Microalgal Biomass

#### 3.2.1. Traditional Folch Method

The Folch extraction method was done following the original procedure described by Folch et al. [51]. A total of 1 g of microalgal biomass was extracted with 20 mL of chloroform:methanol (2:1) vortexing for 2 min. The mixture was centrifuged at 3000 rpm for 10 min and the organic layer was collected. The extraction process was carried out 3 times on the same biomass. The collected organic layers were purified by washing with water and centrifuged at 4000 rpm for 10 min. Finally, the chloroform layer contained the extracted lipids. Samples were evaporated in a rotary evaporator (Heidolph Hei-Vap Value HB/G3, Schwabach, Germany) under reduced pressure at 40 °C. The lipid content was determined gravimetrically and was calculated as a weight percentage of dry biomass. Lipid extracts obtained were stored in dark vessels with an argon atmosphere at 4 °C until their analysis.

#### 3.2.2. Ultrasound-Assisted Extraction

UAE was carried out with an ultrasound bath (Elmasonic P 30H, Elma Schmidbauer GmbH, Singen, Germany) with automatic control of time and temperature. Extractions were done by using an ultrasound frequency of 37 kHz and ultrasonic power of 100 W. Dried microalgal biomass were dispersed in ethanol at a ratio of 1:10 (*w*/*v*). Different experiments were carried out by using different parameters like temperature (30, 40, and 50 °C), and time (15 and 30 min). After the treatment, samples were filtrated, evaporated, and treated as previously described for the other extraction methods.

#### 3.2.3. Enzyme-Based Extraction

The enzymatic pre-treatment of microalgae was done following the procedure previously described by Castejón and Señoráns [10] with some modifications. Briefly, 1 g of dry microalgae biomass was dispersed in 10 mL of acetate buffer (pH 4.5) and different commercial enzymatic solutions (Viscozyme^®^ L, Celluclast^®^ 1.5 L, Saczyme^®^ Yield, and a mix of the enzymatic solutions (1:1:1 *v*/*v*)) were added with an enzyme dosage of 15 mg of protein per gram of biomass [12]. The mixture was incubated at 50 °C with constant shaking (500 rpm) for 1 h by using an Eppendorf Thermomixer C (Hamburg, Germany). Then, the samples were centrifuged at 4000 rpm for 10 min. The enzymatic solution was removed and the pretreated biomass was used for lipid extraction by using ultrasounds (see Section 3.2.1) for 30 min at 50 °C. A control experiment without an enzymatic solution was also done. After the treatment, samples were filtrated, evaporated, and treated as previously described for the other extraction methods.

### 3.3. Fatty Acid Composition by GC-MS

Fatty acid composition of all microalgal extracts was analyzed by GC–MS by using an Agilent 7890A connected to an Agilent 5975C Inert XL EI/CI MSD (Palo Alto, CA, USA). Prior to analysis, fatty acid methyl esters (FAMEs) were freshly prepared by base-catalyzed methanolysis of the glycerides (KOH in methanol). FAMEs were separated by using an HP-5ms ultra-inert column (30 m × 250 µm × 0.25 µm) (Palo Alto, CA, USA). A total of 1 µL of the sample was injected in spitless mode and an injector temperature of 280 °C. The initial oven temperature was set at 150 °C for 1 min and the temperature was gradually raised to 220 °C at 3 °C/min with a final increase to 300 °C for 3 min. Helium was used as carrier gas at a constant column flow rate of 2.52 mL/min. The GC to MS interface temperature was fixed at 280 °C and an electron ionization system was set on the MS in scan mode. The mass range evaluated was 50–600 *m*/*z*, where the MS quad and source temperatures were maintained at 150 °C and 230 °C respectively. Fatty acids were identified by comparing their retention times and mass spectrum profiles with known standards (FAME mix supelco) and the NIST mass spectral library (Version 2.2).

### 3.4. Cell Culture and Treatment

The human colon cancer cell line HCT-116 were purchased from the American Type Culture Collection (ATCC). The epithelial nontumorigenic immortalized cell line HCEC-1CT was kindly provided by Professor Jerry W. Shay (UT Southwestern Medical Center, Dallas, TX, USA). HCT-116 cells were cultivated in Dulbecco’s Modified Eagle Medium (DMEM) GlutaMAX^TM^ medium supplemented with 10% heat-inactivated fetal calf serum (FCS) and 1% *v*/*v* penicillin (5000 units/mL)/streptomycin (5000 µg/mL) (P/S). HCEC-1CT cells were cultivated in high-glucose DMEM combined with 10X medium 199 (2%) and supplemented with cosmic calf serum (2%), HEPES 20 mM, gentamycin (50 µg/mL), insulin–transferrin–selenium-G supplement (10 µl/mL), recombinant human EGF (20 ng/mL), and hydrocortisone (1 µg/mL). Both cell lines were cultivated in humidified incubators (95% humidity) at 37 °C and 5% CO_2_ and regularly tested for mycoplasm.

Test compounds were dissolved in DMSO and added to the incubation media at different concentrations (0.1–200 µg/mL), resulting in a final concentration of 0.5% (*v*/*v*) DMSO for all experiments.

### 3.5. Cell Viability Assays

#### 3.5.1. CellTiter-Blue (CTB) Assay

The CellTiter-Blue (CTB) assay was used to assess changes in the proportion of viable cells. It uses the indicator dye resazurin to measure the metabolic capacity of cells. Briefly, 22,000 HCT-116 cells per well or 15,000 HCEC-1CT cells per well were seeded in 96-well plates and allowed to attach for 24 h. Thereafter, cells were incubated with solvent control (0.5% DMSO) and respective concentrations of the omega-3 lipid extracts for 24 h. After incubation, the medium was removed, and a 1:10 dilution of CellTiter-Blue solution in DMEM medium was added for 1 h. Then, fluorescent signals (excitation 560 nm, emission 590 nm) of supernatants were measured by utilizing a Synergy H1 hybrid multimode reader (BioTek, Bad Friedrichshall, Germany).

#### 3.5.2. Sulforhodamine B (SRB) Assay

The sulforhodamine B assay (SRB assay) was performed according to a modified method of Skehan et al. [52]. The assay measures the optical density (absorbance) of cellular protein stained with the dye sulforhodamine B, showing a high linear correlation between protein content and optical density. Briefly, 22,000 HCT-116 cells per well or 15,000 HCEC-1CT cells per well were seeded in 96-well plates and allowed to attach for 24 h. Thereafter, cells were incubated with solvent control (0.5% DMSO) and respective concentrations of the omega-3 lipid extracts for 24 h. After incubation, cells were fixed by addition of trichloroacetic acid and subsequently stained with SRB solution (0.4% *w*/*v* in 1% acetic acid). After washing with water and 1% *v*/*v* acetic acid, the color was eluted with Tris buffer (10 mM, pH 10). Absorbance was measured at 570 nm with a Synergy H1 hybrid multimode reader (Biotek, Bad Friedrichshall, Germany).

#### 3.5.3. Lactate Dehydrogenase (LDH) Assay

A lactate dehydrogenase (LDH) leakage assay was carried out by using the CyQUANT™ LDH Cytotoxicity Assay kit (Invitrogen™, Waltham, MA, USA). The assay was performed according to manufacturer instructions. Briefly, 22,000 HCT-116 cells per well were seeded in 96-well plates and allowed to grow for 24 h prior to incubation. Thereafter, cells were incubated with solvent control (0.5% DMSO) and respective concentrations of the omega-3 lipid extracts for 24 h. A maximum LDH release was determined by lysing the cells by using the lysis buffer provided by the kit (Triton-X100) for 45 min. After the incubation time, 50 µl of the supernatant of the treated cells was transferred to a fresh 96-well plate and mixed with 50 µl of the LDH reaction mixture. The plate was incubated at room temperature and protected from light for 30 min. Then, the reaction was stopped by adding 50 µl of the stop solution and the absorbance was measured at 490 nm and 680 nm with a Synergy H1 hybrid multimode reader (Biotek, Bad Friedrichshall, Germany) to determine LDH activity.

### 3.6. Dichlorofluorescein (DCF) Assay

The formation of ROS was quantified fluorometrically by using DCFH-DA following the method described by Wang and Joseph [53]. Briefly, 35,000 HCT-116 cells per well were seeded in 96-well plates and allowed to attach for 24 h. Cells were incubated with DCFH-DA for 15 min at 37 °C and washed two times with PBS prior incubation with solvent control (0.5% DMSO) and respective concentrations of the omega-3 lipid extracts for 90 min. After excitation at λ = 485 nm DCF emits light at λ = 528 nm, which was detected with a Synergy H1 hybrid multimode reader (Biotek, Bad Friedrichshall, Germany). The emitted light directly reflects the ROS production in the cells. Fluorescence was detected every 15 min over a period of 90 min. H_2_O_2_ (1 mM) was used as a positive control.

### 3.7. Protective Dichlorofluorescein (pDCF) Assay

A modified version of the DCF assay was used to assess the ability of microalgal lipids to strengthen the antioxidant defense of cells and thus offer protection from H_2_O_2_-induced ROS production. HCT-116 cells (22,000 cells per well) were incubated with the omega-3 lipid extracts for 24 h prior to oxidative stress induction by 1 mM H_2_O_2_ for up to 90 min. ROS levels were determined as described above with the DCF assay.

### 3.8. Statistical Analysis

All extraction experiments were performed in triplicate and the results were expressed as mean ± standard deviation. The effect of extraction method and extraction conditions on the lipid recovery were analyzed by using one-way ANOVA followed by Tukey post-hoc test (differences were considered statistically significant at *p* < 0.05).

Presented data for the cell-based assays are the means + SD of at least three independent biological replicates. Statistical differences compared to the solvent were calculated with a one-sample Student’s *t*-test (*p* < 0.05, *p* < 0.01 and *p* < 0.001). Significant differences among the test concentrations were calculated by using one-way ANOVA followed by Tukey post-hoc test (results were considered as statistically different at *p* < 0.05).

Outliers were eliminated from raw data of experiments with more than five replicates by using the Nalimov test. Statistical analysis was performed with OriginPro 2021 (9.8.0.200) (OriginLab Corporation, Northampton, MA, USA).

## 4. Conclusions

This work provides relevant results for new eco-friendly approaches using enzyme-based alternatives in combination with ultrasounds to extract omega-3-rich lipids from microalgae, avoiding the use of toxic and hazardous solvents. Amongst the different commercial enzymatic solutions investigated, Saczyme showed a high lipid recovery comparable to the traditional Folch method, without affecting the fatty acid profile. Moreover, our preliminary study of cell viability suggests that the omega-3 lipid extract from *Nannochloropsis gaditana* has a selective effect, acting only on the human colon carcinoma cells but not against the nontumorigenic cells. These results may open new possibilities for the green production of bioactive ingredients from microalgae with potential health benefits and applications in the food and nutraceutical industry.

## Figures and Tables

**Table 1:** Fatty acid composition determined by GC–MS of the lipid extracts obtained from *Nannochloropsis gaditana* comparing different extraction techniques.

Fatty Acid	RT (min)	% Fatty Acids					
		Folch	Control	Viscozyme	Celluclast	Saczyme	Mix
*12:0*	6.2	0.4 ± 0.0	0.6 ± 0.1	0.5 ± 0.0	0.5 ± 0.1	0.4 ± 0.1	0.6 ± 0.0
*14:0*	10.0	4.3 ± 0.1	4.5 ± 0.1	4.3 ± 0.1	4.4 ± 0.1	4.3 ± 0.2	4.4 ± 0.1
*16:1 n-7*	14.6	26.1 ± 0.6 ^a^	22.9 ± 0.8 ^b^	24.6 ± 0.7 ^a,b^	24.0 ± 0.8 ^a,b^	24.9 ± 1.1 ^a,b^	23.2 ± 0.6 ^b^
*16:0*	15.1	23.0 ± 0.8 ^a^	18.3 ± 1.3 ^b^	21.7 ± 1.3 ^a,b^	19.7 ± 1.6 ^a,b^	21.5 ± 1.7 ^a,b^	19.1 ± 0.7 ^a,b^
*17:1*	16.3	0.7 ± 0.0	0.7 ± 0.2	0.5 ± 0.2	0.7 ± 0.1	0.5 ± 0.3	0.3 ± 0.1
*18:2 isomer n.i.*	17.1	0.7 ± 0.1	0.5 ± 0.2	0.7 ± 0.1	0.6 ± 0.1	0.7 ± 0.0	0.7 ± 0.0
*18:2 n-6*	19.7	3.2 ± 0.1 ^b^	3.5 ± 0.0 ^a^	3.5 ± 0.0 ^a^	3.5 ± 0.0 ^a^	3.5 ± 0.0 ^a^	3.5 ± 0.0 ^a^
*18:1 n-9*	19.8	4.8 ± 0.1	4.7 ± 0.0	4.7 ± 0.0	4.8 ± 0.0	4.8 ± 0.0	4.7 ± 0.1
*18:1 n-7*	20.0	0.7 ± 0.1	0.6 ± 0.0	0.5 ± 0.1	0.6 ± 0.0	0.5 ± 0.1	0.5 ± 0.0
*18:0*	20.6	0.2 ± 0.0 ^a^	0.2 ± 0.0 ^b^	0.2 ± 0.0 ^b^	0.2 ± 0.0 ^b^	0.2 ± 0.0 ^b^	0.2 ± 0.0 ^b^
*20:4 n-6*	24.2	5.9 ± 0.7 ^b^	7.1 ± 0.0 ^a^	6.8 ± 0.1 ^a,b^	7.0 ± 0.2 ^a^	6.8 ± 0.2 ^a,b^	7.3 ± 0.1 ^a^
*20:5 n-3*	24.5	29.3 ± 0.8	34.5 ± 1.9	30.6 ± 1.9	32.7 ± 2.1	30.2 ± 2.4	33.8 ± 1.1
*20:5 isomer n.i.*	24.6	0.6 ± 0.2	0.9 ± 0.1	1.1 ± 0.1	1.1 ± 0.2	1.1 ± 0.5	1.2 ± 0.4
*20:5 isomer n.i.*	25.8	n.d.	1.1 ± 0.1	0.6 ± 0.2	0.7 ± 0.4	0.6 ± 0.2	0.4 ± 0.2
SFA		27.9 ± 1.0 ^a^	23.5 ± 1.3 ^b^	26.5 ± 1.4 ^a,b^	24.7 ± 1.7 ^a,b^	26.4 ± 1.9 ^a,b^	24.3 ± 0.8 ^a,b^
MUFA		32.4 ± 0.9 ^a^	28.9 ± 1.2 ^b^	30.4 ± 1.1 ^a,b^	30.0 ± 1.1 ^a,b^	30.8 ± 1.7 ^a,b^	28.8 ± 1.2 ^b^
PUFA		39.7 ± 2.0 ^b^	47.6 ± 2.5 ^a^	43.1 ± 2.5 ^a,b^	45.6 ± 2.8 ^a,b^	42.8 ± 3.6 ^a,b^	47.0 ± 1.7 ^a^
n-3		29.3	34.5	30.6	32.7	30.2	33.8
n-6		9.1	10.6	10.3	10.5	10.2	10.8
n-6/n-3 ratio		0.3	0.3	0.3	0.3	0.3	0.3

RT, retention time; SFA, saturated fatty acids; MUFA, monounsaturated fatty acids; PUFA, polyunsaturated fatty acids; n.i., not identified; n.d., not detected. Results expressed as percentage over the total content (relative content). Values are the mean ± SD of three determinations. Different letters indicate statistically significant differences between extraction methods at *p* < 0.05 (one-way ANOVA with post-hoc Tukey, a–b).

**Table 2:** Characteristics, components, and activities of the enzymatic solutions used ^a^.

Product Name	Component Name	Side Activities	Activity	pH Range	T ^a^ Range
Viscozyme^®^ L	Beta-glucanase (endo-1,3(4)-)	xylanase, cellulase and hemicellulase	100 FBG/g	3.3–5.5	40–50 °C
Celluclast^®^ 1.5 L	Cellulase	no reported	700 EGU/g	4.0–6.0	50–60 °C
Saczyme^®^ Yield	Glucoamylase (glucan 1,4-alpha-glucosidase)	alpha-amylase, cellulase, beta-glucosidase, cellulose and 1,4-beta-cellobiosidase	900 AGU/g	3.5–5.5	30–60 °C

^a^ Information provided by the manufacturer.

## Data Availability

The data that support the findings of this study are available from the corresponding author upon reasonable request.
